# Body composition monitoring in children and adolescents: reproducibility and reference values

**DOI:** 10.1007/s00431-021-03936-0

**Published:** 2021-01-22

**Authors:** Annelies Van Eyck, Sofie Eerens, Dominique Trouet, Eline Lauwers, Kristien Wouters, Benedicte Y. De Winter, Johanna H. van der Lee, Koen Van Hoeck, Kristien J. Ledeganck

**Affiliations:** 1 Laboratory of Experimental Medicine and Pediatrics and member of the Infla-Med Centre of Excellence, University of Antwerp, Wilrijk, Belgium; 2 Department of Pediatrics, Antwerp University Hospital, Edegem, Belgium; 3 Clinical Trial Center (CTC), CRC Antwerp, Antwerp University Hospital, Edegem, Belgium; 4 Faculty of Medicine and Health Sciences, University of Antwerp, Antwerp, Belgium; 5 Department of Gastroenterology and Hepatology, Antwerp University Hospital, Edegem, Belgium; 6 Emma Children’s Hospital, Amsterdam UMC, University of Amsterdam, Pediatric Clinical Research Office, AMC, Amsterdam, The Netherlands; 7 University of Antwerp, Universiteitsplein 1, T3.34, 2610 Antwerp, Belgium

**Keywords:** Body composition, Children, Adolescents, Reference, Reproducibility, Reliability

## Abstract

There is an increasing need for suitable tools to evaluate body composition in paediatrics. The Body Composition Monitor (BCM) shows promise as a method, but reference values in children are lacking. Twenty children were included and measured twice by 4 different raters to asses inter- and intra-rater reproducibility of the BCM. Reliability was assessed using the Bland-Altman method and by calculating intraclass correlation coefficients (ICCs). The intra-rater ICCs were high (≥ 0.97) for all parameters measured by BCM as were the inter-rater ICCs for all parameters (≥ 0.98) except for overhydration (0.76). Consequently, a study was set up in which BCM measurements were performed in 2058 healthy children aged 3–18.5 years. The age- and gender-specific percentile values and reference curves for body composition (BMI, waist circumference, fat mass and lean tissue mass) and fluid status (extracellular and intracellular water and total body water) relative to age were produced using the GAMLSS method for growth curves.

*Conclusion:* A high reproducibility of BCM measurements was found for fat mass, lean tissue mass, extracellular water and total body water. Reference values for these BCM parameters were calculated in over 2000 children and adolescents aged 3 to 18 years.

**Table:** 

**What is Known** *• The 4-compartment model is regarded as the ‘gold standard’ of body composition methods, but is inappropriate for regular follow-up or screening of large groups, because of associated limitations.*	
*• Body Composition Monitor® is an inexpensive field method that has the potential to be an adequate monitoring tool.* **What is New** *• Good reproducibility of BCM measurements in children provides evidence to use the device in longitudinal follow-up, multicentre and comparative studies.* *• Paediatric reference values relative to age and sex for the various compartments of the body are provided.*

**Supplementary Information:**

The online version contains supplementary material available at 10.1007/s00431-021-03936-0.

## Introduction

The World Health Organization recognizes childhood obesity as one of the most serious public health challenges of the twenty-first century [1]. Excessive adipose tissue is associated with significant health consequences later in life, such as cardiovascular diseases, type 2 diabetes mellitus and cancer [1]. Therefore, there is an increasing need for tools suitable in paediatric care to evaluate body composition in both clinical and research settings [2]. In addition, the importance of nutritional assessment is not restricted to children with overweight and obesity, but is also highly relevant in a variety of conditions and chronic diseases. Among others, childhood cancer, chronic inflammatory diseases, chronic kidney disease and cystic fibrosis have all been associated with an altered body composition [3].

Different techniques are available for body composition analysis, ranging from basic anthropometric measurements to densitometry and advanced imaging techniques. Body mass index (BMI) is one of the most simple and widely used methods to identify children and adolescents with excess adiposity and malnutrition, but this measure cannot be used to distinguish fat mass from lean tissue mass [3]. This disadvantage is highly relevant in the paediatric population, since the proportion of fat and lean tissue mass during childhood varies by age, gender, ethnicity and the level of hormonal maturation [4]. The 4-compartment (4C) model is the most accurate in vivo measurement of fat mass and lean tissue mass and as such is regarded as the ‘gold standard’ of body composition methods [5]. The 4C model, however, is inappropriate for regular follow-up or screening of large groups of patients, because of its associated limitations such as its high cost and it being a time-consuming procedure [6]. In contrast, the Body Composition Monitor® (BCM, Fresenius Medical Care, Germany) is an inexpensive field method that has the potential to be an adequate monitoring tool [7]. The technology uses bioimpedance spectroscopy (BIS) to determine total body water (TBW) and extracellular water (ECW) volume. Using advanced models, three body composition compartments can be derived from these measurements: lean tissue, adipose tissue and excess fluid or ‘overhydration’ [8]. Parameters of the BCM model have shown good to excellent agreement with the respective gold standard methods in adults [9], but validation studies in children are lacking. To date, only parameters related to the fluid status have been validated in healthy children and children receiving haemodialysis [10]. Initially, the application of the device focused on the evaluation of the fluid status in end-stage renal disease patients. Nevertheless, over the past few years, the BCM is increasingly being used for nutritional assessment as well [11, 12]. The BCM model has shown potential in various populations in paediatrics, but several barriers still need to be overcome to allow a widespread application of the device in both research and clinical practices. More particularly, knowledge of the reproducibility and appropriate reference values are important to help facilitate the interpretation of longitudinal data and results obtained in different centres. Therefore, the aims of this study were firstly to investigate the inter- and intra-rater reproducibility of the BCM device and secondly to provide gender-specific reference curves for body composition relative to age in children aged 3–18 years using the BCM.

## Materials and methods

### Study population

In this prospective study, children aged 3–18 years were recruited between June 2012 and March 2020 through their respective schools.

The ethics committee of the Antwerp University Hospital approved this study (EC n° B300201939802 and B300201214619) and informed consent was obtained from the children and their parents or legal guardians.

### Study design

#### Inter- and intra-rater reproducibility

Twenty healthy children were recruited voluntarily through advertisement in the staff of the Antwerp University Hospital and measurements were performed at the Antwerp University Hospital. Eight investigators from eight different paediatric nephrology centres performed the measurements. A short training was given at the start, including a 15-min teaching DVD with general instructions. Each measurement lasted approximately 5 min. Each child was measured twice by the same investigator with a time interval of 30 min and was measured by the four different investigators in a time frame of four hours. Data of measurements were not visible to other raters. Between measurements, each child was allowed to play for 30 min. During the 4 h of assessment, the children were having free drinks and food. The food and fluid intake was not registered.

#### Reference values

A total of 2058 children were recruited in 15 Belgian schools (see acknowledgments). School management approved the presence of the research team and the study protocol beforehand. Measurements were performed at the school and children were measured consecutively during the school day.

### Anthropometry

Height was measured to the nearest millimetre using a standing stadiometer and weight was recorded to the nearest 0.1 kg using an electronic scale (M302000-02 ADE, Hamburg, Germany). Body mass index (BMI) was calculated as weight (in kilogrammes) over height (in meters) squared and was further analysed as standard deviation scores (SDS), using the Flemish growth study as a reference population [13]. The waist circumference was measured as the smallest circumference between the lowest ribs and highest hip comb approximately 1 cm above the umbilicus.

### Bioimpedance spectroscopy

A BCM measurement was performed in all children (Fresenius Medical Care, St. Wendel, Germany) with the child lying supine.

Electrodes were attached following the wrist-ankle approach, in a tetrapolar arrangement. Four electrodes are used per measurement. Two electrodes are placed on the hand: one on the wrist and the second on the dorsal side of the metacarpalia, close to the phalanges. The other two electrodes are placed on the foot: one on the ankle and the second on the dorsal side of the metatarsals, close to the phalanges of the toes. To guarantee a good contact of the electrodes with the skin, degreasing with diethylether was performed before the placement of the electrodes. Age, gender, height, weight and blood pressure were registered in the device before starting the measurement. If the quality calculated by BCM was < 60%, the measurement was repeated and only good quality measurements were used.

All guidelines for the use of the BCM were followed: non-electrical bed, no cell phones and no electrical devices within 1 m of the device.

The parameters of interest were extracellular water (ECW), intracellular water (ICW) and total body water (TBW) in litres and fat (FM) and lean tissue mass (LTM) in kilogrammes. Overhydration (OH) (in litres) is calculated from the distribution of water in LTM and adipose tissue mass (ATM).

### Statistical analysis

#### Inter- and intra-rater variability

The reproducibility was assessed by calculating the intraclass correlation coefficients (ICCs) according to the following formulas [14] based on the values of the variance components that are derived from analysis of variance (ANOVA) [15]:

For intra-rater reproducibility:

$$ \mathsf{ICC}=\left[{\mathsf{Var}}_{\mathsf{c}}+{\mathsf{Var}}_{\mathsf{c}\ast \mathsf{r}}\right]/\left[{\mathsf{Var}}_{\mathsf{c}}+{\mathsf{Var}}_{\mathsf{m}}+{\mathsf{Var}}_{\mathsf{c}\ast \mathsf{r}}+{\mathsf{Var}}_{\mathsf{c}\ast \mathsf{m}}+{\mathsf{Var}}_{\mathsf{m}\ast \mathsf{r}}+{\mathsf{Var}}^{\mathsf{E}}\right] $$

And for inter-rater reproducibility:

$$ \mathsf{ICC}=\left[{\mathsf{Var}}_{\mathsf{c}}+{\mathsf{Var}}_{\mathsf{c}\ast \mathsf{m}}\right]/\left[{\mathsf{Var}}_{\mathsf{c}}+{\mathsf{Var}}_{\mathsf{r}}+{\mathsf{Var}}_{\mathsf{c}\ast \mathsf{r}}+{\mathsf{Var}}_{\mathsf{c}\ast \mathsf{m}}+{\mathsf{Var}}_{\mathsf{m}\ast \mathsf{r}}+{\mathsf{Var}}_{\mathsf{E}}\right] $$

with Var_c_ as the children-induced variance, Var_r_ as the rater-induced variance and Var_m_ as the measurement-induced variance. Var_c*r_, Var_c*m_ and Var_m*r_ are the variances caused by the interaction of children, raters and measurements, and Var_E_ is the residual or error variance. The ICC ranges from 0 to 1, with higher values indicating a higher reproducibility.

The magnitude of the measurement error expressed as smallest detectable change (SDC) was calculated as SDC = 1.96 * √2 * √VarE and denotes the smallest change within an individual that can be interpreted as ‘real’ change, not due to measurement error [16].

Bland-Altman plots were used to further visualize and quantify inter- and intra-rater reproducibility. For the intra-rater variability, the differences between 2 measurements per combination of child and investigator were plotted against the means of these 2 measurements [17]. Bias and limits of agreement (LoA) (calculated as the mean difference ± 1.96 times the standard deviation of the differences per patient) were calculated. These limits indicate how large the difference between two measurements of the same rater on the same child can be. For the inter-rater variability, we look at modified Bland-Altman plots for multiple observers [18]. The difference between each observation and the mean of all observations for one child are plotted against the overall mean for that child, using a different symbol for each rater. The limits of agreement now represent how different ratings of an individual observer are, compared to the mean of all observers.

Sample size calculation of the reproducibility study was based on the accuracy of the ICC. With an anticipated ICC of 0.85 and a desired precision of 0.1, we needed to include 20 patients in a design with 4 raters [19]. Precision is hereby defined as half width of the 95% confidence interval.

#### Reference values

Gender-specific smoothed centile curves were computed for body composition (BMI, waist circumference, FM, LTM) and fluid status (ECW, ICW and TBW) using generalized additive models for location, scale and shape (GAMLSS) [20]. The GAMLSS method is an extension of the LMS method for modelling growth curves [21] and involves two processes: (i) fitting a parametric distribution for the response variable for each age and (ii) smoothing the distribution across age for each parameter of the selected parametric distribution function. The two processes are estimated simultaneously by iterative penalized likelihood maximization using the R package ‘gamlss’ (http://www.gamlss.org/) [22] in R version 3.6.3 (R Foundation for Statistical Computing, Vienna, Austria, http://cran.us.r-project.org/). Within the GAMLSS framework, we assessed three distribution families: Box-Cox Cole and Green (BCCG), Box-Cox power exponential (BCPE) and Box-Cox t (BCT). These distribution families allow the modelling of the location (mu), variance (sigma), skewness (nu) and depending on the distribution kurtosis (tau). Goodness of fit of the models was assessed using worm plots, Q-statistic and visual inspection of the centile curves. Model selection was based on the generalized Akaike information criterion (G-AIC) and goodness of fit. Final GAMLSS model parameters were used to produce centile tables and curves for boys and girls separately.

Precision of the centile curves is estimated based on bootstrap simulations in analogy with Cole et al. [23] In 1000 bootstrap runs, the standard error for the different percentiles is computed relative to the age-dependent standard deviation.

## Results

### Inter- and intra-rater variability

A total of 20 children were included with a median age of 9 (3–17) years, a median weight of 30 (15–65) kg, and a median height of 1.35 (0.99–1.77) m. The median BMI was 16.6 (13.9–24.6) kg/m^2^, which corresponds to a BMI SDS of − 0.1 (− 1.3–1.8). All measurements were successful and were performed within the allotted time. None of the measurements was excluded from further analysis.

**Fig. 1:**
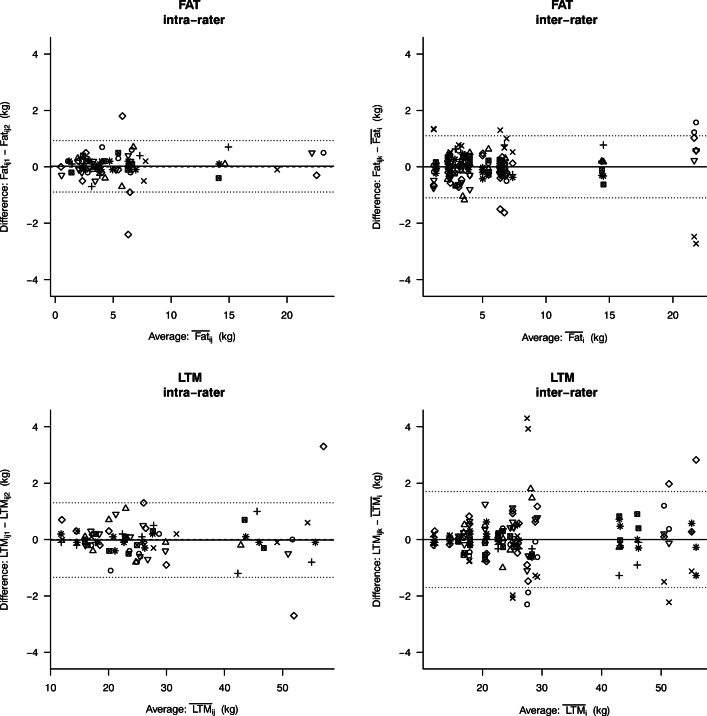
Bland-Altman plots for fat mass (FAT) and lean tissue mass (LTM). The left panels show classical Bland-Altman plots for intra-rater reproducibility with two repeated measures per child-rater combination. The right panels show modified Bland-Altman plots for inter-rater reproducibility with 4 raters. Each rater is depicted by a different symbol. The mean of the within-person differences and upper and lower limits of agreement are depicted by the horizontal lines

**Fig. 2:**
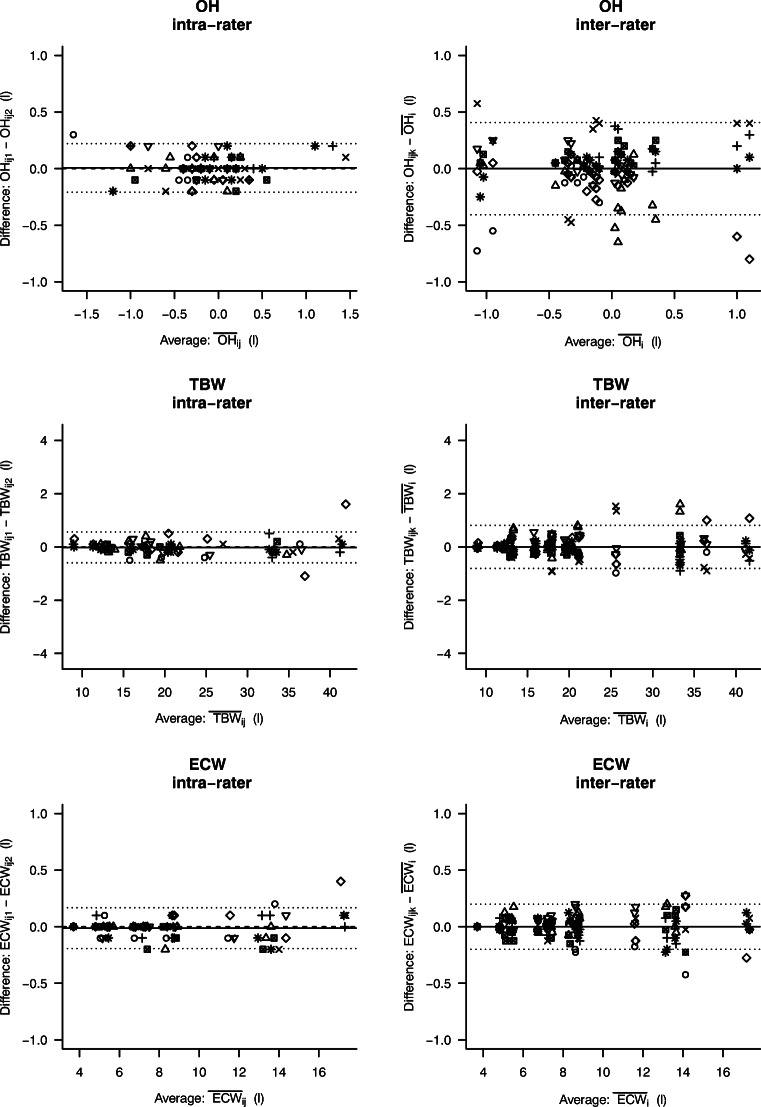
Bland-Altman plots for overhydration (OH), total body water (TBW) and extracellular water (ECW). The left panels show classical Bland-Altman plots for intra-rater reproducibility with two repeated measures per child-rater combination. The right panels show modified Bland-Altman plots for inter-rater reproducibility with 4 raters. Each rater is depicted by a different symbol. The mean of the within-person differences and upper and lower limits of agreement are depicted by the horizontal lines

The reproducibility data according to the Bland-Altman analysis, SDC and intra- and inter-observer ICCs are shown in Table 1**.** Bland-Altman plots for all parameters are shown in Figs. 1 and 2**.** No systematic difference was found between the pairs of measurements by the same rater.

**Table 1:** Reproducibility in terms of intraclass correlation coefficients, Bland-Altman analysis and smallest detectable change

	ICC		Bland-Altman analysis			SDC
			Intra-rater		Inter-rater	
	Intra- observer	Inter- observer	Mean difference	LoA	LoA	
FAT (kg)	0.99	0.98	0.02	− 0.89–0.93	− 1.10–1.10	0.92
LTM (kg)	0.99	0.99	− 0.02	− 1.34–1.30	− 1.70–1.70	1.34
TBW (litres)	0.99	0.99	− 0.02	− 0.60–0.56	− 0.81–0.81	0.58
ECW (litres)	0.99	0.99	− 0.01	− 0.19–0.17	− 0.20–0.20	0.17
OH (litres)	0.97	0.76	0.006	− 0.21–0.22	− 0.41–0.41	0.23

*ICCs* intraclass correlation coefficients, *LoA* limits of agreement, *SDC* smallest detectable change, *LTM* lean tissue mass, *TBW* total body water, *ECW* extracellular water, *OH* overhydration

### Reference values

A total of 2119 children and adolescents were included into the study, of whom ten were excluded due to a failed measurement, three children refused the BCM measurement, three children were excluded for comorbidities (chronic diseases) and 45 were excluded for not fulfilling the inclusion criteria: 22 children were younger than 3 years and 23 adolescents were older than 18 years. In total, 2058 subjects were included for further analysis, with a mean age of 11 ± 4 years and 51% of the population was male. The average weight was 40.9 ± 18.8 kg and the average height was 145.2 ± 24.9 cm, which corresponds with an average BMI of 18.2 ± 3.5 kg/m^2^ and BMI SDS of – 0.05 ± 0.95.

A wide range of BMI SDS was apparent across all age ranges, as is shown in Supplemental Fig. S1.

**Fig. 3:**
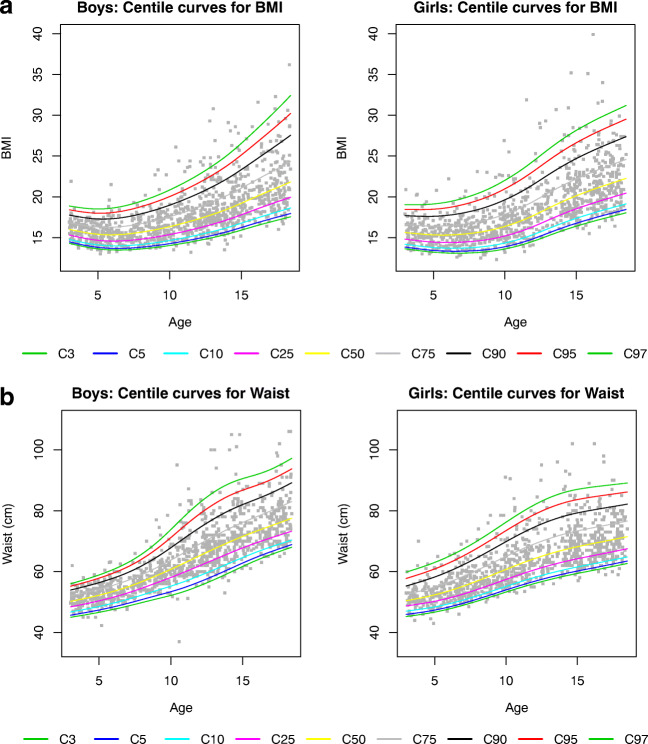

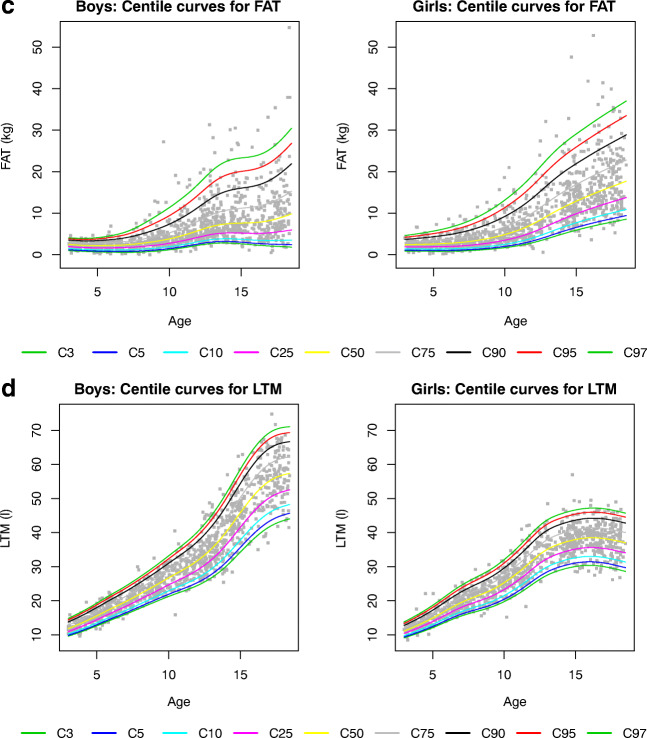
Percentile graphs for age in boys (left panels) and girls (right panels) aged 3 to 18 years. **a** Body mass index (BMI), **b** waist, **c** fat mass (FAT) and **d** lean tissue mass (LTM)

Supplemental Tables S1–S4 show the gender- and age-specific percentile values for BMI, waist circumference, FM and LTM at the 3th, 5th, 10th, 25th, 75th, 90th, 95th and 97th percentiles. Figure 3 shows the corresponding reference curves. BMI, waist circumference, FM and LTM increased with age in both genders.

At the 50th percentile, the FM for boys is 2.48 kg at 3 years and increases to 9.22 kg at 18 years, while for girls, the FM starts at 2.39 kg but increases to a median of 17.06 kg at the age of 18 years. The opposite can be seen in LTM, with a higher median increase with age in boys compared to girls (11.84 kg to 56.96 kg vs. 11.14 to 37.50 kg at 18 years, respectively). This gender difference extrapolates to all percentiles (Supplemental Table). In contrast, BMI did not show this discrepancy, as the median BMI for boys is 16.02 kg/m^2^ at 3 years and increases to 21.50 kg/m^2^ at 18 years, while the median BMI for girls starts at 15.63 kg/m^2^ and increases to 21.97 kg/m^2^ at 18 years.

**Fig. 4:**
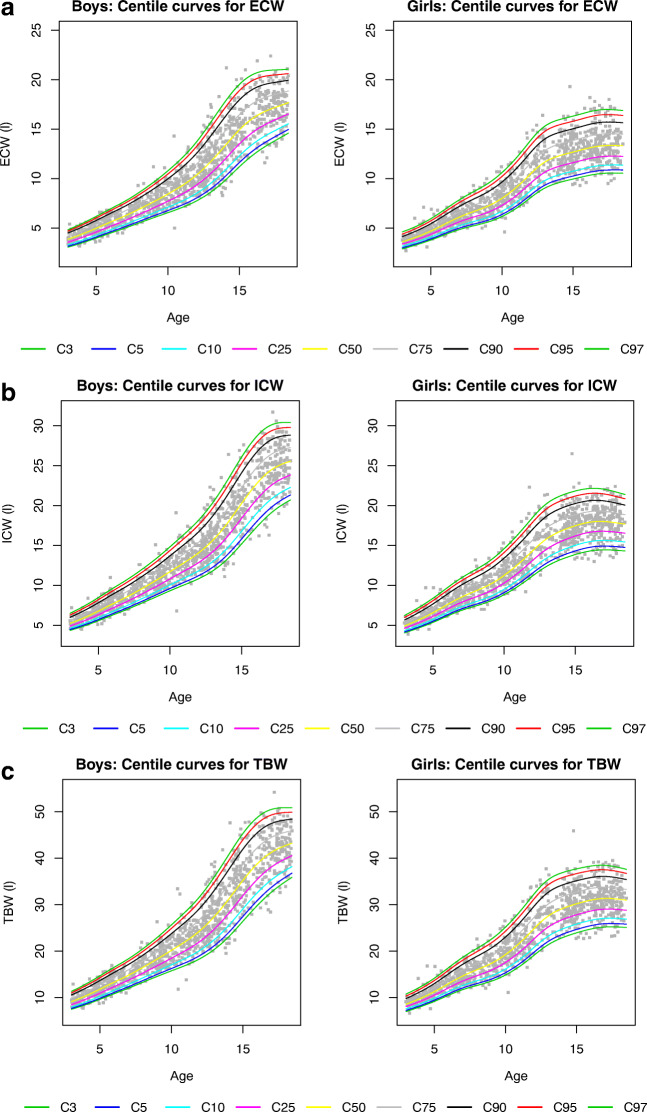
Percentile graphs for age in boys (left panels) and girls (right panels) aged 3 to 18 years. **a** Extracellular water (ECW), **b** intracellular water (ICW) and **c** total body water

Gender- and age-specific percentile values for ECW, ICW and TBW at the 3th, 5th, 10th, 25th, 75th, 90th, 95th and 97th percentiles are shown in Supplemental Tables S5–S7 with the corresponding reference curves in Fig. 4. ECW, ICW and TBW all increased with age in both genders; however, this increase was more pronounced in boys compared to girls for all parameters.

The precision of the percentile curves, as computed by bootstrap simulations, is between 10 and 15% of the age-dependent standard deviation in the middle range of the ages and 30% in the boundaries of the sample.

## Discussion

Body composition measurements are gaining interest in the scientific scene of chronic diseases [24–26] as well as childhood obesity [27, 28]. Reliable tools for measuring the various compartments of the body are therefore essential in order to perform research in a standardized manner in the most optimal circumstances. Besides, reference values for each body compartment are needed to draw the right conclusions when measuring the body composition in children and adolescents who suffer from obesity or other chronic diseases. In the first part of this manuscript, the reproducibility and reliability of BCM measurements were demonstrated. In the second part, reference curves for BCM were plotted in over 2000 Belgian children aged 3 to 18 years.

Bioimpedance analysis (BIA) such as BCM® type BIS-BIA by Fresenius is a relatively simple, quick and non-invasive technique that is easy to use when performing field studies involving children [29]. The intra- and inter-rater reliability of BCM was obtained in twenty children who were each measured twice by four different raters. The applicability of the Fresenius device in clinical studies was confirmed with exclusively successful measurements without the necessity for any repeated measurements. In this study, a high intra- and inter-rater reproducibility of the BCM measurements in healthy children aged 3 to 18 years (ICC ≥ 0.98) was observed for fat mass, lean tissue mass, extracellular water and total body water, making the Fresenius BCM a reliable tool for the monitoring of individual prospective changes in hydration status, fat mass and lean tissue mass in longitudinal studies. Besides, since the observer-dependent bias remained low, we provide evidence that the BCM device is indeed useful for multicentre and comparative studies. However, as can be noticed in the Bland-Altman figures, there are some divergent measurements for each body compartment without any reasonable explanation. The same BCM device and investigation room were used by the co-raters, and the quality of each measurement was rated at more than 75%.

For ‘overhydration’, the intra-rater agreement between measurements was high (ICC 0.97); however, the inter-rater reproducibility did not perform so well (ICC 0.76). ‘Overhydration’ should be disregarded in healthy children [10] as it might depend on fluid intake and physical activity. The children included into this study were measured by four different raters. Each rater performed two measurements in the same child within 30 min. Given this short time between measurements, no influence of fluid intake was expected in the child and the intra-rater agreement performed well. However, there was a longer period between the measurements of the different observes (1 to 4 h) and the children had free access to fluids during the entire study protocol, potentially explaining the higher inter-rater variability for overhydration. Therefore, the relatively low inter-rater reproducibility is considered an artefact as a result of a design flaw.

Based on the reproducibility data, reference values in children and adolescents were calculated for each separate body compartment, except for ‘overhydration’. First, anthropology was verified with the measurements of weight, length, BMI (and BMI SDS) and waist circumference. BMI corrected for age and sex is still considered a proxy of adiposity in children and adolescents [30]. However, BMI does not distinguish between fat mass and lean tissue mass and does thus not accurately reflect the body composition [31]. In the present population, BMI z-scores were similar to the reference values described by Roelants et al [13]. The children and adolescents included in the present study thus represent the overall population. In contrast to BMI, waist circumference is a better estimate of trunk fat mass in children and adolescents [32]. In comparison with the reference values in 580 New Zealand children and adolescents [32], in 22,000 Brazilian children aged 6 to 10 years [33] and in Tunisian children [34], the waist circumference was slightly lower in each age category in our population, confirming the need for reference values at a regional level. The waist circumference values of the children aged 3 to 10 years are highly comparable to the European values published by Nagy et al. in 2014 [31]. In our population, in addition to the 3- to 10-year-old children, also reference values are calculated for children aged 10 to 18 years and might thus serve as reference values in a European population.

Second, BCM measurements were performed. To date, BCM is mostly applied to estimate the body fluid overload in dialysis patients [10]. For this purpose, the device is well validated in adults and children [10, 35]. However, reference values for fat mass and lean tissue mass in children and adolescents are lacking. In the present study, over 2000 children and adolescents were included in 16 elementary and high schools spread over Flanders and Brussels. With the increasing interest in body composition studies in obesity and chronic diseases, the results described in this manuscript might serve as reference values for future studies on this topic.

Fat mass or adipose tissue is a major storage for energy and besides actively secretes metabolically active adipokines involved in among others inflammatory processes [36]. An accurate measurement of the fat mass is thus essential in research projects investigating the adipose tissue in health and disease. In children and adolescents with intentional (overweight, obesity) or unintentional weight loss (acute or chronic diseases), it is of utmost interest to make a distinction between a reduction in fat mass or in a reduction in muscle mass (protein-energy wasting syndrome). Since the BMI is not able to discriminate fat mass from lean tissue mass, the BCM is a superior alternative by differentiating both fat mass and lean tissue mass. Our main goal was to provide reference values for fat mass and lean tissue mass measured by the Fresenius BCM device. In this study, the association of fat mass with age was less steep in boys than in girls. At the age of 18 years, the median fat mass was 1.85 times higher in girls compared to boys. The opposite was seen in lean tissue mass, with a higher median increase with age in boys compared to girls. This gender difference extrapolates to all percentiles. When compared to the reference study of Nagy et al [31], where fat mass was calculated based on skin folds, the difference in median fat mass between boys and girls at the age of 10 years was comparable to our results (factor 1.22 and 1.27, respectively). In a British population, the difference between boys and girls was more pronounced at the age of 10 years (factor 1.5 in girls compared to boys), while this difference was less visible at the age of 18 years (factor 1.5 in the British population) [37].

In this paper, reference values for extracellular water and total body water are provided as well. The generalizability of total body water measured by the Fresenius BCM device was validated in children and adolescents in the Dasgupta study [10]. Extracellular water, intracellular water and total body water all increased with age in both genders; however, this increase was more pronounced in boys compared to girls for all parameters. Food and drink intake of the children was not monitored in this study. However, according to Wabel et al. [38], food intake does not influence the reliability of the device. Since overhydration is supposed to be absent in healthy children, no reference values for this parameter were calculated. From the statistical analysis, however, it was clear that overhydration was not age dependent (data not shown).

The major strength of this study was the combination of reproducibility of the BCM results on the one hand and the inclusion of over 2000 patients with age range between 3 and 18 years on the other hand, resulting in reliable reference values. The data published in this manuscript therefore serve as a solid reference base for future multicentre or longitudinal studies investigating body composition in children and adolescents.

Unfortunately, we were not able to include children younger than 3 years and reference body composition values in this very young population remain therefore unexplored. Furthermore, it should be kept in mind that BIS is not a direct measurement of the body compartments but only of the electrical properties of tissues that are used for the calculation of body composition [39]. A volume model based on the Hanai mixture theory is used to determine TBW, ECW and ICW [35]. Overhydration, LTM and adipose tissue are then calculated by a body composition algorithm from the ECW and TBW information [8].

In conclusion, this paper provides evidence for the reproducibility and reliability of BCM measurements with excellent intra- and inter-rater reproducibility for fat mass, lean tissue mass, extracellular water and total body water. Reference values for these BCM parameters were calculated in over 2000 Belgian children and adolescents aged 3 to 18 years.

## Supplementary Information

ESM 1 (PDF 718 kb)

## Abbreviations

ATM: Adipose tissue mass
ANOVA: Analysis of variance
BCM: Body Composition Monitor®
BIS: Bioimpedance spectroscopy
BMI: Body mass index
ECW: Extracellular water
FM: Fat mass
GAMLSS: Generalized additive models for location, scale and shape
ICC: Intraclass correlation coefficients
ICW: Intracellular water
LoA: Limits of agreement
LTM: Lean tissue mass
OH: Overhydration
SDC: Smallest detectable change
SDS: Standard deviation score
TBW: Total body water
