# Supplementary material for: Body composition monitoring in children and adolescents: reproducibility and reference values
Source: Eur J Pediatr. 2021 Jan 22;180(6):1721–32. doi: 10.1007/s00431-021-03936-0 (PMC8105252; doi:10.1007/s00431-021-03936-0)
Supplement: Supplementary file 1 — (PDF 718 kb) [file 431_2021_3936_MOESM1_ESM.pdf]

Body composition monitoring in children and adolescents: reproducibility and reference values

A. Van Eyck and S. Eerens, D. Trouet, E. Lauwers, K. Wouters, B.Y. De Winter, J.H. van der Lee , K. Van Hoeck and K.J. Ledeganck*

***** Laboratory of Experimental Medicine and Pediatrics and member of the Infla-Med Centre of Excellence, University of Antwerp, Wilrijk, Belgium.

E-mail address: kristien.ledeganck@uantwerp.be

Online supplemental material for the European Journal of Pediatrics

Figure S1: Body mass index SDS (BMI SDS) distribution per age

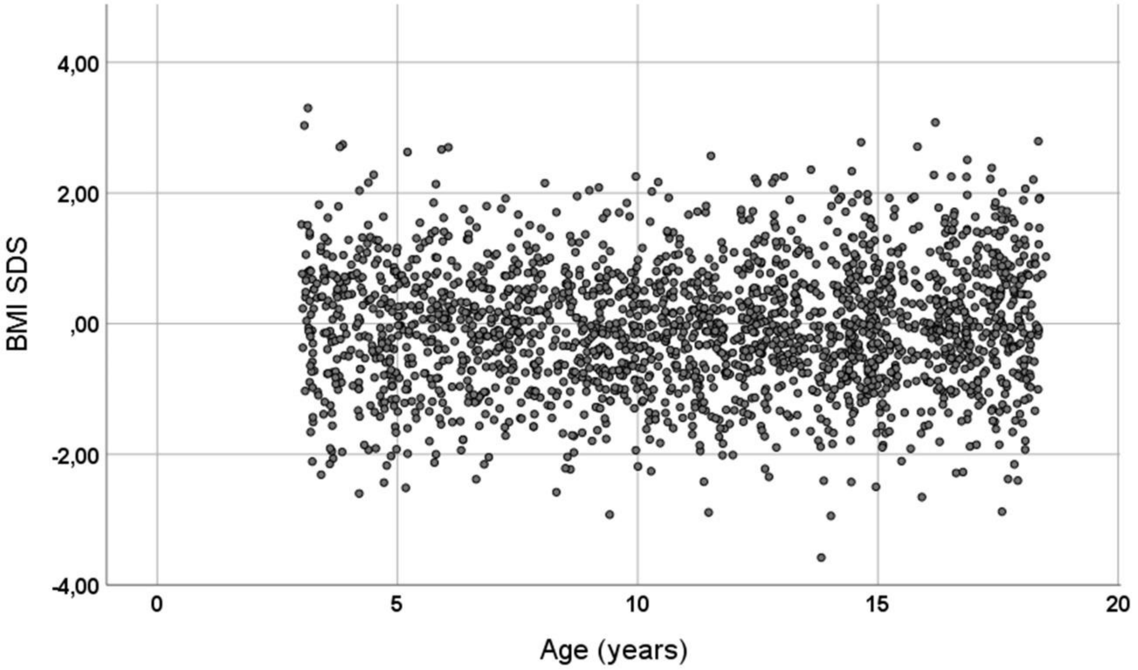

Table S1: Body mass index (BMI) distribution by percentiles in boys (upper panel) and girls (lower panel) aged 3 to 18 years.

BMI – BOYS

Age	C3	C5	C10	C25	C50	C75	C90	C95	C97
**3**	14.30	14.48	14.78	15.32	16.02	16.86	17.77	18.41	18.88
**4**	13.91	14.09	14.39	14.94	15.66	16.52	17.46	18.13	18.62
**5**	13.62	13.80	14.11	14.67	15.41	16.30	17.29	17.99	18.51
**6**	13.48	13.68	13.99	14.57	15.34	16.27	17.31	18.06	18.62
**7**	13.49	13.69	14.02	14.63	15.43	16.42	17.53	18.34	18.94
**8**	13.61	13.82	14.16	14.80	15.66	16.71	17.91	18.79	19.45
**9**	13.79	14.01	14.37	15.05	15.96	17.09	18.39	19.35	20.08
**10**	14.03	14.26	14.64	15.37	16.34	17.56	18.97	20.03	20.83
**11**	14.32	14.56	14.97	15.74	16.79	18.10	19.65	20.81	21.71
**12**	14.64	14.90	15.33	16.16	17.28	18.71	20.40	21.69	22.69
**13**	15.01	15.28	15.75	16.63	17.84	19.40	21.25	22.69	23.81
**14**	15.42	15.72	16.21	17.17	18.48	20.17	22.22	23.82	25.08
**15**	15.92	16.23	16.77	17.80	19.22	21.07	23.34	25.14	26.57
**16**	16.43	16.77	17.34	18.45	20.00	22.03	24.55	26.57	28.19
**17**	16.91	17.27	17.89	19.08	20.76	22.98	25.77	28.03	29.88
**18**	17.36	17.75	18.41	19.69	21.50	23.92	27.01	29.55	31.64

BMI – GIRLS

Age	C3	C5	C10	C25	C50	C75	C90	C95	C97
**3**	13.63	13.84	14.18	14.81	15.63	16.61	17.70	18.47	19.03
**4**	13.40	13.61	13.96	14.61	15.45	16.48	17.62	18.44	19.04
**5**	13.21	13.43	13.78	14.45	15.33	16.41	17.61	18.48	19.12
**6**	13.11	13.33	13.70	14.40	15.32	16.45	17.73	18.67	19.37
**7**	13.10	13.33	13.71	14.44	15.41	16.62	18.00	19.02	19.78
**8**	13.16	13.40	13.81	14.57	15.60	16.89	18.39	19.50	20.35
**9**	13.32	13.58	14.00	14.82	15.91	17.30	18.92	20.15	21.08
**10**	13.61	13.88	14.33	15.19	16.37	17.86	19.63	20.98	22.02
**11**	14.01	14.30	14.78	15.70	16.96	18.57	20.49	21.97	23.12
**12**	14.56	14.86	15.37	16.36	17.70	19.44	21.52	23.13	24.39
**13**	15.19	15.52	16.06	17.11	18.55	20.41	22.65	24.38	25.74
**14**	15.82	16.17	16.74	17.85	19.38	21.35	23.72	25.57	27.02
**15**	16.39	16.75	17.36	18.52	20.12	22.19	24.68	26.62	28.14
**16**	16.90	17.28	17.91	19.12	20.79	22.94	25.53	27.54	29.11
**17**	17.37	17.76	18.41	19.67	21.39	23.62	26.30	28.37	29.98
**18**	17.82	18.22	18.89	20.19	21.97	24.26	27.02	29.14	30.79

Table S2: Waist distribution by percentiles in boys (upper panel) and girls (lower panel) aged 3 to 18 years.

WAIST – BOYS

Age	C3	C5	C10	C25	C50	C75	C90	C95	C97
**3**	44.98	45.67	46.71	48.41	50.19	52.03	53.92	55.16	56.00
**4**	45.77	46.49	47.59	49.35	51.17	53.07	55.08	56.43	57.36
**5**	46.62	47.39	48.55	50.39	52.25	54.21	56.37	57.86	58.90
**6**	47.58	48.41	49.64	51.59	53.50	55.54	57.87	59.53	60.72
**7**	48.67	49.56	50.90	52.98	54.97	57.11	59.68	61.57	62.95
**8**	49.85	50.84	52.31	54.58	56.72	59.04	61.96	64.18	65.84
**9**	51.01	52.11	53.76	56.29	58.67	61.27	64.70	67.40	69.47
**10**	52.18	53.40	55.22	58.07	60.75	63.75	67.84	71.18	73.81
**11**	53.58	54.87	56.83	59.94	62.96	66.4	71.19	75.19	78.42
**12**	55.33	56.64	58.65	61.92	65.21	69.06	74.41	78.92	82.61
**13**	57.32	58.59	60.58	63.9	67.42	71.61	77.34	82.15	86.11
**14**	59.46	60.67	62.59	65.87	69.55	73.99	79.89	84.78	88.76
**15**	61.68	62.80	64.60	67.78	71.55	76.14	82.01	86.74	90.54
**16**	63.77	64.80	66.48	69.53	73.36	78.09	83.86	88.37	91.91
**17**	65.63	66.58	68.16	71.13	75.08	80.03	85.84	90.26	93.66
**18**	67.32	68.21	69.71	72.64	76.79	82.10	88.15	92.64	96.06

WAIST – GIRLS

Age	C3	C5	C10	C25	C50	C75	C90	C95	C97
**3**	45.24	45.94	47.01	48.73	50.43	52.43	55.27	57.75	59.82
**4**	45.90	46.58	47.64	49.42	51.33	53.60	56.67	59.23	61.34
**5**	46.70	47.36	48.41	50.24	52.37	54.94	58.23	60.89	63.02
**6**	47.72	48.37	49.41	51.31	53.66	56.55	60.09	62.85	65.03
**7**	48.95	49.59	50.65	52.63	55.21	58.43	62.24	65.15	67.40
**8**	50.33	50.98	52.06	54.13	56.95	60.52	64.64	67.75	70.12
**9**	51.80	52.46	53.58	55.76	58.82	62.74	67.22	70.56	73.11
**10**	53.29	53.98	55.14	57.43	60.73	64.99	69.84	73.46	76.22
**11**	54.73	55.45	56.65	59.06	62.57	67.14	72.37	76.26	79.24
**12**	56.08	56.82	58.07	60.58	64.27	69.11	74.64	78.78	81.96
**13**	57.30	58.06	59.36	61.95	65.78	70.80	76.57	80.88	84.20
**14**	58.41	59.19	60.51	63.17	67.08	72.22	78.11	82.51	85.89
**15**	59.40	60.18	61.52	64.21	68.17	73.37	79.27	83.66	87.01
**16**	60.31	61.10	62.45	65.15	69.13	74.32	80.17	84.47	87.73
**17**	61.24	62.03	63.38	66.08	70.06	75.22	80.97	85.15	88.30
**18**	62.19	62.98	64.33	67.03	71.00	76.12	81.76	85.81	88.83

Table S3: Fat mass distribution by percentiles in boys (upper panel) and girls (lower panel) aged 3 to 18 years.

FAT (KG) – BOYS

Age	C3	C5	C10	C25	C50	C75	C90	C95	C97
**3**	1.01	1.20	1.49	1.96	2.48	3.01	3.51	3.83	4.05
**4**	0.81	1.00	1.28	1.75	2.27	2.81	3.34	3.69	3.94
**5**	0.69	0.88	1.17	1.66	2.20	2.79	3.39	3.81	4.11
**6**	0.61	0.83	1.15	1.66	2.26	2.92	3.65	4.20	4.63
**7**	0.58	0.82	1.17	1.74	2.40	3.18	4.11	4.88	5.52
**8**	0.65	0.91	1.31	1.96	2.72	3.67	4.92	6.06	7.09
**9**	0.84	1.12	1.55	2.28	3.18	4.35	6.00	7.61	9.14
**10**	1.20	1.48	1.94	2.75	3.81	5.27	7.37	9.41	11.37
**11**	1.70	1.99	2.48	3.41	4.70	6.54	9.17	11.65	13.93
**12**	2.27	2.58	3.12	4.19	5.79	8.12	11.40	14.37	16.97
**13**	2.73	3.07	3.67	4.92	6.85	9.72	13.76	17.31	20.33
**14**	2.83	3.20	3.85	5.25	7.44	10.74	15.31	19.25	22.52
**15**	2.63	3.02	3.72	5.23	7.62	11.19	16.04	20.07	23.31
**16**	2.33	2.75	3.51	5.15	7.76	11.59	16.63	20.67	23.83
**17**	2.10	2.57	3.42	5.29	8.25	12.53	17.98	22.19	25.40
**18**	1.93	2.48	3.48	5.70	9.22	14.22	20.40	25.04	28.50

FAT (KG) – GIRLS

Age	C3	C5	C10	C25	C50	C75	C90	C95	C97
**3**	0.89	1.09	1.39	1.87	2.39	2.99	3.67	4.20	4.63
**4**	0.86	1.07	1.38	1.89	2.49	3.17	3.97	4.61	5.11
**5**	0.83	1.04	1.37	1.92	2.59	3.38	4.33	5.09	5.70
**6**	0.84	1.05	1.39	1.99	2.73	3.66	4.79	5.71	6.45
**7**	0.92	1.14	1.50	2.15	2.99	4.08	5.44	6.57	7.50
**8**	1.09	1.32	1.71	2.44	3.42	4.70	6.37	7.77	8.94
**9**	1.35	1.61	2.05	2.88	4.03	5.58	7.62	9.36	10.83
**10**	1.71	2.01	2.52	3.49	4.86	6.75	9.24	11.38	13.18
**11**	2.21	2.57	3.17	4.33	5.98	8.25	11.26	13.83	15.99
**12**	2.94	3.37	4.08	5.49	7.49	10.24	13.84	16.87	19.38
**13**	3.91	4.42	5.29	6.98	9.37	12.63	16.84	20.32	23.15
**14**	4.96	5.55	6.56	8.51	11.25	14.94	19.62	23.41	26.46
**15**	5.92	6.58	7.70	9.87	12.90	16.93	21.94	25.94	29.10
**16**	6.77	7.50	8.72	11.09	14.39	18.73	24.05	28.23	31.50
**17**	7.53	8.32	9.64	12.21	15.77	20.42	26.06	30.42	33.80
**18**	8.21	9.06	10.49	13.25	17.06	22.01	27.94	32.47	35.95

Table S4: lean tissue mass (LTM) distribution by percentiles in boys (upper panel) and girls (lower panel) aged 3 to 18 years.

LTM (KG) – BOYS

Age	C3	C5	C10	C25	C50	C75	C90	C95	C97
**3**	9.60	9.85	10.25	10.96	11.84	12.80	13.76	14.38	14.80
**4**	11.01	11.30	11.76	12.58	13.57	14.66	15.73	16.42	16.89
**5**	12.65	12.98	13.51	14.44	15.57	16.81	18.01	18.78	19.30
**6**	14.37	14.74	15.34	16.41	17.68	19.06	20.40	21.25	21.82
**7**	16.03	16.45	17.13	18.31	19.73	21.25	22.72	23.65	24.27
**8**	17.80	18.28	19.04	20.38	21.97	23.67	25.30	26.32	27.01
**9**	19.56	20.11	20.98	22.50	24.30	26.22	28.05	29.20	29.96
**10**	21.27	21.90	22.91	24.66	26.73	28.92	31.01	32.32	33.19
**11**	22.80	23.53	24.69	26.71	29.08	31.59	33.97	35.45	36.44
**12**	24.48	25.33	26.67	29.00	31.74	34.63	37.35	39.05	40.17
**13**	26.80	27.80	29.38	32.13	35.33	38.69	41.86	43.82	45.11
**14**	30.11	31.30	33.17	36.39	40.14	44.05	47.71	49.96	51.45
**15**	34.32	35.70	37.86	41.59	45.88	50.33	54.46	57.00	58.67
**16**	38.46	40.00	42.41	46.52	51.23	56.08	60.55	63.27	65.06
**17**	41.67	43.30	45.85	50.17	55.07	60.08	64.66	67.45	69.27
**18**	43.54	45.19	47.76	52.09	56.96	61.90	66.40	69.11	70.88

LTM (KG) – GIRLS

Age	C3	C5	C10	C25	C50	C75	C90	C95	C97
**3**	9.14	9.41	9.80	10.43	11.14	11.92	12.76	13.36	13.81
**4**	10.53	10.85	11.31	12.07	12.92	13.85	14.84	15.55	16.07
**5**	12.12	12.49	13.04	13.94	14.97	16.08	17.25	18.08	18.68
**6**	13.91	14.35	15.00	16.08	17.30	18.63	20.01	20.97	21.68
**7**	15.55	16.05	16.81	18.07	19.49	21.03	22.63	23.73	24.52
**8**	16.79	17.36	18.21	19.62	21.23	22.96	24.74	25.95	26.82
**9**	18.01	18.64	19.59	21.16	22.94	24.86	26.81	28.13	29.07
**10**	19.75	20.46	21.53	23.29	25.30	27.44	29.60	31.05	32.07
**11**	22.07	22.87	24.08	26.08	28.34	30.74	33.14	34.73	35.85
**12**	24.90	25.79	27.15	29.39	31.91	34.57	37.20	38.93	40.13
**13**	27.48	28.45	29.91	32.32	35.03	37.86	40.62	42.42	43.66
**14**	29.08	30.08	31.59	34.07	36.84	39.72	42.50	44.29	45.53
**15**	30.07	31.09	32.63	35.16	37.98	40.88	43.66	45.44	46.66
**16**	30.40	31.44	33.02	35.61	38.48	41.42	44.21	45.98	47.19
**17**	30.02	31.09	32.71	35.35	38.26	41.24	44.04	45.80	47.00
**18**	29.14	30.23	31.87	34.55	37.50	40.49	43.29	45.04	46.22

Table S5: extracellular water (ECW) distribution by percentiles in boys (upper panel) and girls (lower panel) aged 3 to 18 years.

ECW (L) – BOYS

Age (y)	C3	C5	C10	C25	C50	C75	C90	C95	C97
**3**	3.07	3.16	3.31	3.56	3.87	4.19	4.50	4.69	4.82
**4**	3.50	3.60	3.77	4.05	4.39	4.75	5.10	5.31	5.46
**5**	3.99	4.10	4.29	4.61	4.99	5.39	5.78	6.02	6.19
**6**	4.50	4.63	4.83	5.19	5.61	6.06	6.50	6.77	6.95
**7**	5.00	5.14	5.36	5.76	6.23	6.73	7.21	7.51	7.71
**8**	5.52	5.68	5.94	6.38	6.92	7.49	8.03	8.38	8.61
**9**	6.04	6.23	6.52	7.03	7.65	8.31	8.95	9.36	9.63
**10**	6.55	6.77	7.11	7.71	8.43	9.22	9.98	10.46	10.79
**11**	7.09	7.34	7.73	8.44	9.29	10.23	11.13	11.71	12.10
**12**	7.74	8.04	8.50	9.34	10.35	11.46	12.55	13.24	13.71
**13**	8.63	8.98	9.53	10.52	11.72	13.03	14.32	15.14	15.70
**14**	9.82	10.21	10.84	11.97	13.33	14.80	16.25	17.16	17.78
**15**	11.20	11.63	12.31	13.50	14.93	16.48	17.96	18.90	19.53
**16**	12.45	12.87	13.56	14.75	16.16	17.65	19.08	19.97	20.57
**17**	13.42	13.83	14.48	15.61	16.92	18.30	19.60	20.40	20.94
**18**	14.25	14.64	15.23	16.27	17.45	18.69	19.84	20.55	21.02

ECW (L) – GIRLS

Age	C3	C5	C10	C25	C50	C75	C90	C95	C97
**3**	2.90	3.02	3.17	3.39	3.61	3.85	4.15	4.40	4.62
**4**	3.32	3.43	3.60	3.84	4.11	4.40	4.73	4.99	5.19
**5**	3.79	3.91	4.09	4.38	4.70	5.06	5.45	5.73	5.95
**6**	4.34	4.47	4.68	5.02	5.41	5.85	6.32	6.65	6.90
**7**	4.85	5.00	5.23	5.63	6.10	6.63	7.19	7.58	7.85
**8**	5.23	5.40	5.66	6.11	6.66	7.28	7.93	8.36	8.68
**9**	5.60	5.79	6.07	6.59	7.21	7.92	8.65	9.15	9.50
**10**	6.17	6.38	6.70	7.29	8.01	8.82	9.66	10.22	10.61
**11**	6.99	7.23	7.60	8.27	9.10	10.04	11.00	11.63	12.08
**12**	8.06	8.32	8.75	9.52	10.46	11.53	12.62	13.34	13.83
**13**	9.03	9.32	9.79	10.63	11.66	12.82	13.99	14.75	15.28
**14**	9.59	9.89	10.37	11.23	12.29	13.47	14.65	15.42	15.95
**15**	9.96	10.26	10.75	11.62	12.68	13.86	15.04	15.80	16.32
**16**	10.29	10.60	11.09	11.98	13.06	14.25	15.43	16.19	16.70
**17**	10.51	10.83	11.33	12.22	13.31	14.51	15.69	16.45	16.97
**18**	10.57	10.88	11.38	12.27	13.35	14.53	15.70	16.44	16.95

Table S6: intracellular water (ICW) distribution by percentiles in boys (upper panel) and girls (lower panel) aged 3 to 18 years.

ICW (L) – Boys

Age	C3	C5	C10	C25	C50	C75	C90	C95	C97
**3**	4.36	4.47	4.63	4.91	5.23	5.60	6.00	6.28	6.50
**4**	4.92	5.05	5.24	5.56	5.95	6.38	6.84	7.17	7.41
**5**	5.58	5.73	5.95	6.33	6.78	7.29	7.83	8.21	8.48
**6**	6.29	6.46	6.72	7.16	7.69	8.27	8.89	9.31	9.62
**7**	7.00	7.18	7.48	7.98	8.57	9.23	9.92	10.39	10.73
**8**	7.76	7.98	8.31	8.88	9.55	10.30	11.06	11.58	11.95
**9**	8.54	8.78	9.17	9.82	10.59	11.45	12.31	12.90	13.31
**10**	9.33	9.61	10.05	10.82	11.72	12.70	13.70	14.37	14.83
**11**	10.07	10.40	10.91	11.80	12.85	13.99	15.14	15.90	16.42
**12**	10.83	11.22	11.82	12.86	14.07	15.40	16.71	17.57	18.17
**13**	11.84	12.29	13.00	14.23	15.66	17.21	18.73	19.71	20.39
**14**	13.28	13.82	14.66	16.09	17.76	19.54	21.26	22.36	23.12
**15**	15.18	15.80	16.76	18.39	20.25	22.21	24.08	25.26	26.06
**16**	17.20	17.87	18.90	20.64	22.61	24.65	26.56	27.75	28.55
**17**	18.97	19.65	20.69	22.42	24.36	26.33	28.15	29.28	30.03
**18**	20.27	20.91	21.89	23.51	25.30	27.10	28.75	29.75	30.41

ICW (L) – Girls

Age	C3	C5	C10	C25	C50	C75	C90	C95	C97
**3**	4.07	4.18	4.35	4.63	4.92	5.26	5.67	5.99	6.24
**4**	4.67	4.80	5.00	5.33	5.69	6.11	6.6	6.97	7.23
**5**	5.37	5.51	5.74	6.13	6.58	7.10	7.68	8.09	8.39
**6**	6.15	6.31	6.57	7.03	7.59	8.22	8.90	9.36	9.70
**7**	6.87	7.05	7.34	7.87	8.53	9.28	10.06	10.58	10.94
**8**	7.42	7.62	7.95	8.54	9.29	10.15	11.02	11.59	11.98
**9**	8.01	8.23	8.60	9.27	10.11	11.07	12.03	12.66	13.09
**10**	8.84	9.10	9.52	10.28	11.24	12.32	13.41	14.11	14.59
**11**	9.90	10.20	10.69	11.58	12.67	13.88	15.10	15.89	16.43
**12**	11.18	11.53	12.10	13.11	14.32	15.66	17.01	17.89	18.49
**13**	12.40	12.80	13.43	14.54	15.83	17.24	18.67	19.61	20.25
**14**	13.24	13.67	14.35	15.50	16.81	18.21	19.65	20.59	21.24
**15**	13.86	14.31	15.01	16.18	17.47	18.84	20.25	21.18	21.82
**16**	14.30	14.76	15.47	16.65	17.91	19.23	20.61	21.51	22.14
**17**	14.46	14.92	15.63	16.78	18.00	19.28	20.59	21.46	22.06
**18**	14.38	14.84	15.53	16.65	17.81	19.02	20.26	21.08	21.64

Table S7: total body water (TBW) distribution by percentiles in boys (upper panel) and girls (lower panel) aged 3 to 18 years.

TBW (L) – BOYS

Age	C3	C5	C10	C25	C50	C75	C90	C95	C97
**3**	7.45	7.63	7.92	8.45	9.09	9.81	10.53	10.99	11.31
**4**	8.45	8.66	9.00	9.60	10.34	11.16	11.98	12.51	12.87
**5**	9.60	9.84	10.23	10.93	11.78	12.72	13.65	14.25	14.65
**6**	10.82	11.10	11.54	12.34	13.30	14.36	15.40	16.06	16.51
**7**	12.01	12.32	12.83	13.72	14.80	15.97	17.12	17.85	18.34
**8**	13.28	13.64	14.22	15.24	16.47	17.80	19.10	19.93	20.48
**9**	14.54	14.96	15.63	16.83	18.26	19.81	21.32	22.27	22.91
**10**	15.74	16.24	17.04	18.45	20.15	21.98	23.76	24.88	25.64
**11**	16.93	17.52	18.45	20.12	22.10	24.25	26.33	27.64	28.52
**12**	18.37	19.06	20.16	22.10	24.41	26.90	29.30	30.80	31.81
**13**	20.36	21.17	22.46	24.74	27.44	30.33	33.09	34.83	35.99
**14**	23.08	24.01	25.50	28.10	31.17	34.42	37.52	39.45	40.73
**15**	26.44	27.48	29.10	31.93	35.24	38.71	41.98	44.00	45.33
**16**	29.82	30.89	32.56	35.45	38.79	42.25	45.48	47.46	48.77
**17**	32.73	33.76	35.37	38.13	41.27	44.51	47.50	49.32	50.51
**18**	35.02	35.97	37.44	39.93	42.75	45.62	48.25	49.85	50.89

TBW (L) – GIRLS

Age	C3	C5	C10	C25	C50	C75	C90	C95	C97
**3**	6.98	7.22	7.55	8.03	8.54	9.10	9.76	10.30	10.74
**4**	8.00	8.25	8.61	9.18	9.79	10.47	11.23	11.81	12.27
**5**	9.17	9.45	9.86	10.52	11.28	12.11	13.01	13.67	14.17
**6**	10.50	10.81	11.29	12.08	13.01	14.03	15.12	15.88	16.44
**7**	11.70	12.05	12.60	13.54	14.65	15.88	17.16	18.04	18.68
**8**	12.61	13.00	13.62	14.70	15.98	17.42	18.89	19.89	20.59
**9**	13.51	13.95	14.65	15.87	17.34	18.98	20.65	21.77	22.55
**10**	14.85	15.35	16.14	17.54	19.24	21.12	23.03	24.29	25.17
**11**	16.71	17.29	18.20	19.81	21.76	23.92	26.09	27.50	28.48
**12**	19.09	19.74	20.77	22.60	24.81	27.23	29.64	31.20	32.26
**13**	21.37	22.07	23.19	25.17	27.54	30.12	32.64	34.27	35.37
**14**	22.88	23.60	24.75	26.76	29.15	31.73	34.23	35.82	36.89
**15**	23.93	24.66	25.81	27.81	30.18	32.71	35.15	36.68	37.70
**16**	24.74	25.47	26.62	28.62	30.97	33.46	35.83	37.31	38.30
**17**	25.18	25.90	27.04	29.02	31.33	33.77	36.07	37.50	38.46
**18**	25.17	25.88	27.00	28.92	31.16	33.50	35.70	37.07	37.97
